# Extracellular and Intracellular Polyphenol Oxidases Cause Opposite Effects on Sensitivity of *Streptomyces* to Phenolics: A Case of Double-Edged Sword

**DOI:** 10.1371/journal.pone.0007462

**Published:** 2009-10-14

**Authors:** Han-Yu Yang, Carton W. Chen

**Affiliations:** Department of Life Sciences and Institute of Genome Sciences, Shih-Pai, Taipei, Taiwan; University of Wisconsin-Milwaukee, United States of America

## Abstract

Many but not all species of *Streptomyces* species harbour a bicistronic *melC* operon, in which *melC2* encodes an extracellular tyrosinase (a polyphenol oxidase) and *melC1* encodes a helper protein. On the other hand, a *melC-*homologous operon (*melD*) is present in all sequenced *Streptomyces* chromosomes and could be isolated by PCR from six other species tested. Bioinformatic analysis showed that *melC* and *melD* have divergently evolved toward different functions. MelD2, unlike tyrosinase (MelC2), is not secreted, and has a narrower substrate spectrum. Deletion of *melD* caused an increased sensitivity to several phenolics that are substrates of MelD2. Intracellularly, MelD2 presumably oxidizes the phenolics, thus bypassing spontaneous copper-dependent oxidation that generates DNA-damaging reactive oxygen species. Surprisingly, *melC^+^* strains were more sensitive rather than less sensitive to phenolics than *melC* ^−^ strains. This appeared to be due to conversion of the phenolics by MelC2 to more hydrophobic and membrane-permeable quinones. We propose that the conserved *melD* operon is involved in defense against phenolics produced by plants, and the sporadically present *melC* operon probably plays an aggressive role in converting the phenolics to the more permeable quinones, thus fending off less tolerant competing microbes (lacking *melD*) in the phenolic-rich rhizosphere.

## Introduction

The ability to produce melanin from tyrosine is found in diverse species of *Streptomyces* [1], [2], [3], and the production of the pigment has been used in taxonomy of the genus. The chain of reactions leading to melanin is initiated by a secreted copper-containing polyphenol oxidase (PPO), ‘tyrosinase’. A typical tyrosinase catalyzes the first two steps of oxidation: insertion of oxygen in a position *ortho* to an existing hydroxyl group of the phenolic substrate (monophenol oxidase activity) followed by oxidation of the diphenol to the corresponding *o*-benzoquinone (diphenol oxidase activity).

The *Streptomyces* tyrosinase is encoded by a bicistronic operon, *melC*. The downstream gene, *melC2*, encodes an apotyrosinase that lacks a signal peptide sequence and remains inactive in the cytosol until it is activated and secreted with the help of the product of the upstream gene, *melC1*. MelC1 is required for copper incorporation, activation, and secretion of the tyrosinase [4], [5], [6]. The *melC* operon has been mapped to the linear chromosomes of many *Streptomyces* species. It is highly unstable, being prone to spontaneous deletion along with neighboring sequences [7], [8], [9], [10]. This reflects a terminal location of this operon on the linear chromosomes [10].

Although melanogenesis was discovered in *Streptomyces* very early and studied extensively, the biological role of tyrosinase in *Streptomyces* remains obscure. The enzyme produces melanin readily in the presence of tyrosine and copper, but tyrosine is neither the best substrate nor the inducer [11]. Production of melanin does not offer protection against UV irradiation on solid medium (our observation), raising doubt that the natural role of *Streptomyces* tyrosinase is melanin production. There are hypotheses that *Streptomyces* tyrosinase may be involved in degradation of lignin [12] and defense against toxic phenolics produced by plants [13]. The *melC* operon of *Streptomyces griseus,* which was shown to cause precocious formation of aerial mycelium on a high-copy-number plasmid in a previous report [14], turned out to be a *melC1-melC2* homologous pair (*griEF*) involved in grixazone biosynthesis [15; see later]. However, this phenomenon is not generally observed, and is complicated by the likely involvement of copper, which can stimulate sporulation [16], [17], [18].

Recently, as the complete genomic sequences for various *Streptomyces* species became available, a bicistronic operon (here designated *melD*) homologous to *melC* has been found in all these sequenced chromosomes. The *melD* operons share the same helper protein (‘*melD1*’)-PPO (‘*melD2*’) organization. Three of these species, *S. avermitilis*, *S. scabies*, and *S. griseus*, which are melanogenic (Mel^+^), also contain a *melC* operon. The fourth species, *S. coelicolor*, does not have a *melC* operon (Mel^−^).

*S. griseus* is unique in possessing, in addition to *melC* and *melD*, another pair of *melC1* and *melC2* homologs (*griE* and *griF*, respectively) embedded in the grixazone biosynthetic gene cluster, which participate in the biosynthesis of this secondary metabolite [15]. Involvement of PPO in the biosynthesis of secondary metabolites is well known in eukaryotes. The *griE-griF* gene pair does not participate in melanogenesis [14], [15].

In this study we found *melD* in all *Streptomyces* species investigated, suggesting that *melD* is more widespread than *melC* in *Streptomyces*, and thus may play a more important biological role. We therefore investigated the function of *melD* in protection against phenolics. MelD2, unlike MelC2, is located intracellularly, and has a narrower substrate specificity. A Δ*melD* mutant exhibited an increased sensitivity to a group of phenolics, which were also substrates for MelD2. This supported the protective role of MelD2 against the toxicity of these phenolics. In contrast, the presence of *melC* increased the sensitivity of *Streptomyces* to the phenolics. Isotope studies indicated that the presence of *melC* caused increased uptake of catechol. Presumably the extracellular MelC2 oxidizes phenolics to more hydrophobic quinones, which enter the mycelium more efficiently. From these results we propose that, in the phenolic-rich rhizosphere, such a detrimental effect of secreted MelC2 is directed toward other competing microbes that lack an intracellular defense system such as *melD*.

## Results

### Occurrence of *melC* and *melD* operons in *Streptomyces* species

**Figure 1:**
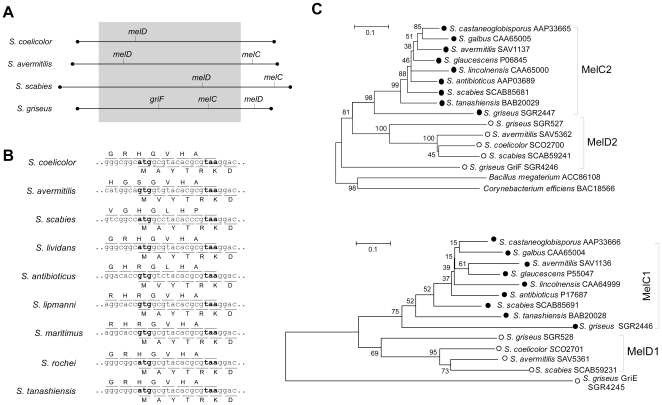
Occurrence of the *melC* and *melD* operons. A. Chromosomal locations. The approximate locations of *melC* and *melD* on the four sequenced *Streptomyces* chromosomes are marked. Filled circles, terminal proteins; gray area, approximate ‘core region’ of the chromosomes. B. Overlapping region of *melD1* and *melD2*. The stop codon of *melD1* and the initiation codon of *melD2* are in bold face. C. Phylogenetic trees of MelC2 (top) and MelC1 (bottom) homologs. The sequences were aligned using ClustalX version 1.8 with the following parameters: method: accurate; matrix: Gonnet; gap open penalty: 10; gap extension penalty: 0.1. The phylogenetic trees were generated using the Neighbor-Joining method. The bootstrap values (in percentages) from 500 reiterations are shown. The capacity to produce melanin is indicated by filled circles, and the inability to produce melanin by open circles.

*melD* is found on the linear chromosomes of four *Streptomyces* species that have been sequenced and annotated so far. Of these, *melC* is found in the three species that are known to produce melanin, *S. avermitilis*, *S. griseus*, and *S. scabies*. In *S. avermitilis* and *S. scabies, melC* is in the terminal regions of the chromosomes, which are known to be unstable, undergoing frequent deletions [19], [20], while *melD* lies in the ‘core region’. In *S. griseus*, *melC* is more centrally located than *melD* (Fig. 1A). In *S. coelicolor*, which lacks *melC*, *melD* is also in the ‘core region’.

The instability of the *melC* operon in *S. avermitilis* was demonstrated by the isolation of 18 melanin-negative (Mel^−^) colonies among 1,700 colonies grown from individual spores. Eleven of the Mel^−^ mutants had lost the *melC* sequence (Southern hybridization data not shown). Such instability of *melC* in *S. avermitilis* is at about the same level as in several other species studied [e.g.], [7], [8], [9,10]. The seven Mel^−^ derivatives still possessing *melC* are presumably defective in other genetic elements required for expression of tyrosinase, as previously described in other species [21], [22], [23]. All 18 Mel^−^ mutants still contained the *melD* sequence (Southern hybridization data not shown), indicating that *melD* is not involved in the production of melanin.

The finding of *melD* on all the sequenced *Streptomyces* chromosomes suggested that it might be widespread. To test this, a pair of primers based on conserved sequences in *melD1* and *melD2* was used to attempt isolation of partial *melD* sequences from six *Streptomyces* species, *S. antibioticus*, *S. lividans*, *S. lipmanii*, *S. maritimus*, *S. rochei*, and *S. tanashiensis,* by PCR. All six yielded the expected sequence spanning the C-terminus (40 aa) of MelD1 and the N-terminus (136–141 aa) of MelD2. All these *melD* sequences, and those from the four sequenced chromosomes, showed highly conserved 17-bp overlaps (spanning five amino acids) between *melD1* and *melD2* (Fig. 1B), except for that in *S. griseus*, in which *melD1* and *melD2* are separated by 19 bp. In contrast, *melC1* and *melC2* do not overlap in the known *melC* operons, except that of *S. lavendulae*, in which the stop codon of *melC1* overlaps the initiation codon of *melC2* [24].

Phylogenetic analysis separated the MelC2 and MelD2 sequences into two distinct branches with similar topologies (Fig. 1C top), and the MelC1 and MelD1 sequences into two others (Fig. 1C bottom). There is a clear correspondence between melanin production (filled circles) and the presence of *melC*. On the other hand, *melD* is present in all species examined regardless of their ability to produce melanin. The topologies of the MelC1/MelD1 and the MelC2/MelD2 phylogenetic trees showed a high degree of congruence (Fig. 1C), indicating that *melC* and *melD* have diversified after duplication of an ancestral bicistronic operon, and that in each operon the helper protein (*melC1*/*melD1*) and the polyphenol oxidase (*melC2*/*melD2*) genes have co-evolved. It is noteworthy that, in the MelC2/MelD2 phylogenetic tree, the evolutionary distances of the MelD2s are significantly longer than those of the MelC2s, indicating divergent evolution of the former away from the latter (see also below). Phylogenetic analysis of the six partial MelD1 (C-terminal 40 aas) and MelD2 sequences (N-terminal 136 aas) also separated them from the MelC1 and MelC2 sequences, respectively (Supporting Information Fig. S1). A tblastn search in the NCBI microbial database revealed 13 more *melD* and five *melC* operons in 12 unannotated *Streptomyces* chromosome sequences. Interestingly, one of these 12 chromosomes (*Streptomyces* sp. C) contains two *melC* and two *melD* operons. The MelC2 and MelD2 sequences encoded by these operons were also distinctly separated in phylogenetic analysis (Supporting Information, Fig. S2).

The *S. griseus* genome is unique in containing a third *melC*-homologous operon, the *griE*-*griF* pair, in the grixazone biosynthetic gene cluster. In the phylogenetic trees, both GriF (MelC2 homolog) and GriE (MelC1 homolog) lie outside the MelC2 and MelD2 and the MelC1 and MelD1 branches, respectively (Fig. 1C). Interestingly, MelC1, MelC2, MelD1, and MelD2 of *S. griseus* also occupy a boundary position within the respective clusters separated by a long evolutionary distance. This hints at a rapid evolution of these homologs in this *S. griseus* strain (IFO 13350, a Waksman and Henrici 1948 strain).

### MelD2 of *S. coelicolor* is located intracellularly and lacks typical tyrosinase activity

MelC1 of *S. antibioticus* contains a Tat signal peptide at the N-terminus [25], and is secreted by the twin-arginine translocation (Tat) pathway [25], a major secretion system in *Streptomyces* [26]. MelC2 is secreted by hitchhiking on MelC1 as a complex [6].

**Figure 2:**
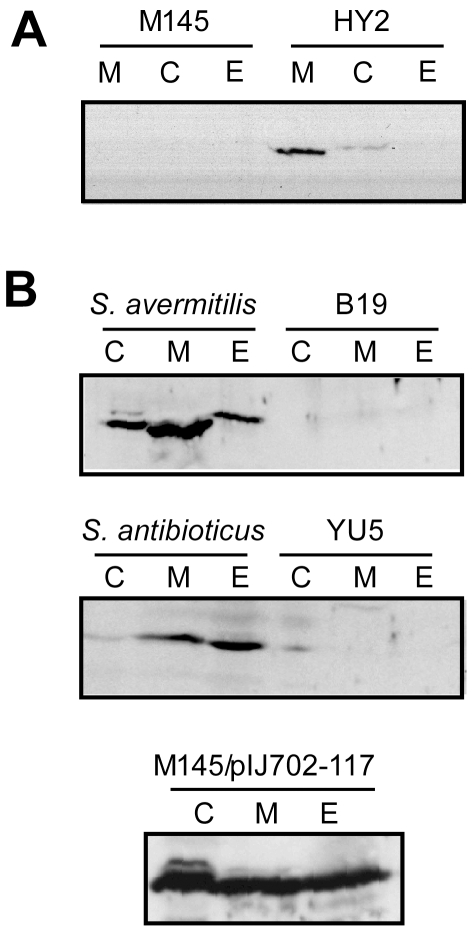
Cellular locations of MelD2 and MelC2. Samples of 10 ml of *Streptomyces* cultures grown to late log phase in TSB were harvested. Cytosolic (C), membrane (M), and extracellular (E) fractions of the cultures were fractionated and the proteins separated by SDS gel electrophoresis. MelD2-His and MelC2 proteins were detected by immunoblotting using anti-polyhistidine (A) and anti-MelC2 (B) antibodies, respectively. B19 is a *ΔmelC* mutant of *S. avermitilis* isolated in this study. YU5 is a *ΔmelC* mutant of *S. antibioticus* isolated previously [10]. M145/pIJ702-117 is M145 harbouring pIJ702-117, a high-copy-number plasmid containing the *melC* operon from *S. antibioticus* under a strong promoter [27].

MelD1 of *S. coelicolor* does not possess a Tat signal peptide. To determine the cellular location of MelD2, a His6 tag was added to the C-terminus of *melD2* on the *S. coelicolor* M145 chromosome. HY2, which contained such His-tagged MelD2, was cultured in TSB liquid medium, harvested, and sonicated. The culture was separated into cytosolic, membrane, and extracellular fractions by centrifugation. Immunoblot using anti-polyhistidine antiserum showed that the His-tagged MelD2 was present mainly in the membrane fraction and nominally in the cytosol, but undetectable extracellularly (Fig. 2A). In comparison, MelC2 was detected by anti-MelC2 antiserum in all three fractions of *S. avermitilis, S. antibioticus*, and M145 harbouring pIJ702-117, containing the *melC* operon from *S. antibioticus* [27] (Fig. 2B). The extracellular presence of MelC2 in these cultures was confirmed by enzymatic assay using DOPA as substrate (data not shown). In contrast, none of the cellular fractions of M145 and HY69 exhibited DOPA oxidization activity, suggesting that DOPA was not a substrate for MelD2.

### Δ*melD* mutants are supersensitive to catechol

**Figure 3:**
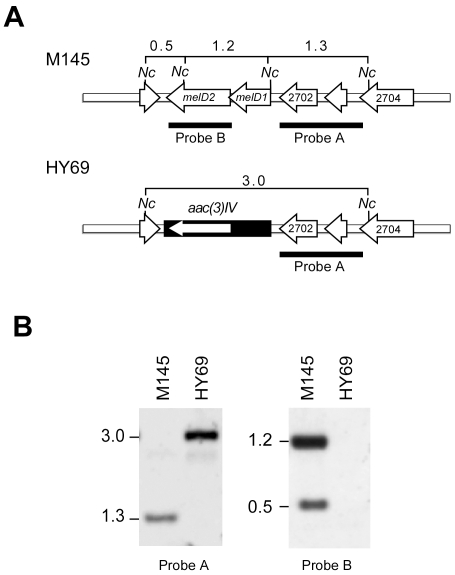
*melD* deletion (Δ*melD*) mutant of *S. coelicolor*. A. Maps of the *melD* operon and the Δ*melD*::*aac(3)IV* mutation. The *melD* operon on the M145 chromosome and the surrounding genes are shown as open arrows. In the Δ*melD*::*aac(3)IV* mutation, the *melD* operon is replaced by the apramycin resistance cassette (filled box) containing *aac(3)IV* (white arrow). The extent of the hybridization probes, A and B, is indicated by the filled boxes. The restriction sites for *Nco*I (Nc) and the sizes of the expected hybridizing fragments are indicated. B. Southern hybridization analysis of the Δ*melD* mutant. Genomic DNA from M145 and HY69 was digested with *Nco*I, separated by electrophoresis, and hybridized with probes A (left panel) and B (right panel).

**Figure 4:**
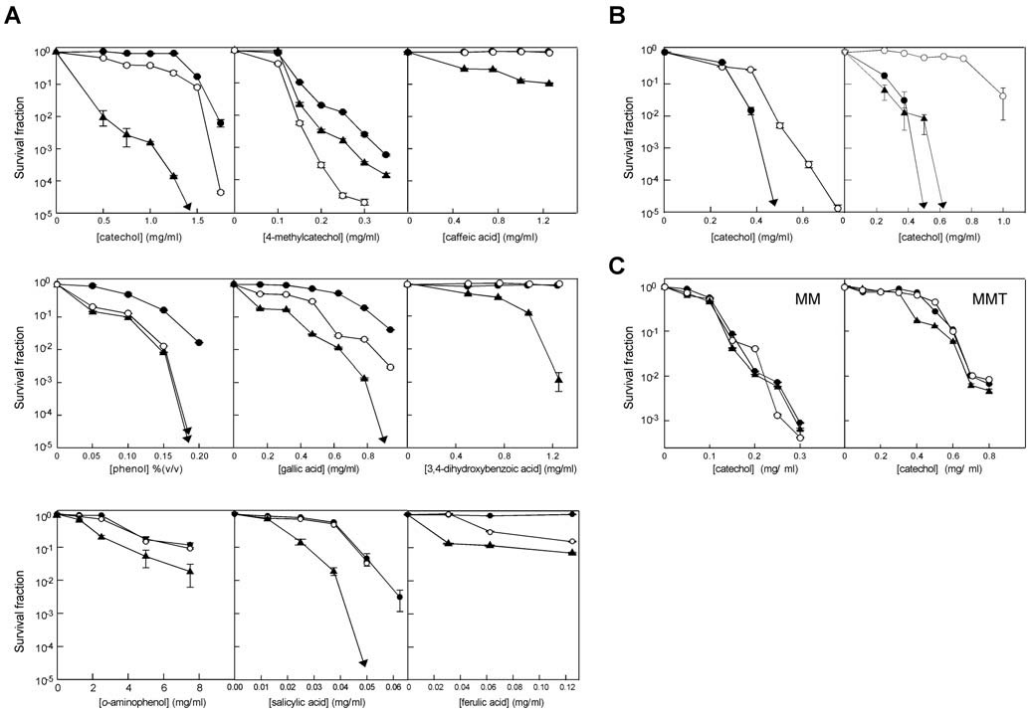
Sensitivity of *Streptomyces* to phenolic compounds. Diluted spore suspensions were plated on medium containing various concentrations of the phenolic compounds and incubated at 30°. After 4 days, the surviving colonies were counted. Survival levels below the lowest scales in the graph are indicated by the arrows. The horizontal bars represent standard deviations. A. Effect of *melC* and *melD* in *S. coelicolor*. Medium: R5. Filled circles, M145; open circles, HY69; filled triangles, M145/pIJ702-117. B. Effect of *melC* in *S. avermitilis* and *S. antibioticus*. Medium: R5. Left panel, *S. avermitilis* (filled circles, wild type; open circles, Δ*melC* mutant B19). Right panel, *S. antibioticus* (filled circles, wild type; open circles, Δ*melC* mutant YU5; filled triangles, YU5-117, a YU5 derivative containing an insert of *melC* from pIJ702-117). C. Effect of media. Left, MM; Right, MMT. The symbols are as in A.

To investigate the biological role of *melD*, a deletion mutation (Δ*melD*) was created in *S. coelicolor* M145 by replacing the *melD* operon by the apramycin resistance gene *aac(3)IV* through homologous recombination (Fig. 3A). A mutant, designated HY69, was isolated, in which replacement of *melD* was confirmed by restriction and hybridization analysis (Fig. 3B). HY69 was tested for increased sensitivity to nine phenolic compounds by counting survivors on R5 medium containing the phenolics (Fig. 4A). Compared to M145, HY69 exhibited an increased sensitivity to catechol, 4-methylcatechol, phenol, gallic acid, and ferulic acid, but not to caffeic acid, 3,4-dihydroxybenzoic acid, *o-*aminophenol, and salicylic acid. These results suggested that *melD* was involved in protection against a specific group of phenolic compounds.

**Figure 5:**
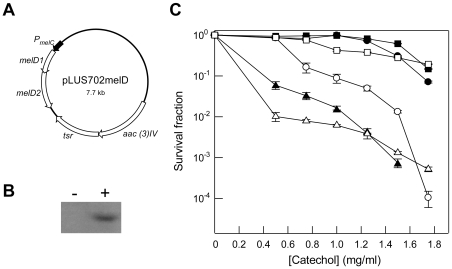
Complementation and additivity studies. A. Complementation vector pLUS702melD. This plasmid is derived from pIJ702-117, the *melC* operon of which was replaced by the *melD* operon of *S. coelicolor* with a C-terminal his6-tagged *melD2* (as in HY2), and the apramycin resistance gene inserted. B. Expression of *melD* on pLUS702melD. An overnight culture of *S. lividans*/pLUS702melD in TSB was collected and sonicated, and the proteins separated by SDS-PAGE. The presence of His6-tagged MelD2 was detected by immunoblot using anti-polyhistidine antiserum. ‘-’ *S. lividans* 1326; ‘+’, *S. lividans* 1326 harbouring pLUS702melD. C. Effect of genetic complementation and additivity. The sensitivity test was performed as in Fig. 4. Filled circles, M145; open circles, HY69; filled triangles, M145/pIJ702-117; open triangles, HY69/pIJ702-117; filled squares, M145/pLUS702melD; open squares, HY69/pLUS702melD. The horizontal bars represent standard deviations.

To complement the *melD* defect in HY69, plasmid pLUS702medD was constructed by substituting the *melC* operon on pIJ702-117 (see below) with the *melD* operon of M145 containing a His6-tagged *melD2* and inserting the apramycin resistance gene (Fig. 5A). pLUS702medD was introduced into HY69 by transformation, and the expression of His6-tagged MelD2 was confirmed by immunoblot using the anti-polyhistidine antibody (Fig. 5B). On R5 medium, HY69/pLUS702melD exhibited the same level of resistance to catechol as M145 (Fig. 5C, open squares). The results indicated that His6-tagged MelD2 was functional and complemented the *melD* defect in HY69. Complementation with a single copy of *melD* (without the His6 tag) inserted into the chromosome of HY69 also restored its catechol resistance to the wild-type level (data not shown).

### *melC* ^+^ strains are supersensitive to phenolic compounds

To test whether *melC* was also involved in resistance to phenolic compounds, M145/pIJ702-117 was compared to M145 for sensitivity to them on R5 medium. Surprisingly, M145/pIJ702-117 was more sensitive to all nine phenolic compounds tested (Fig. 4A, filled triangles). The test was extended to *S. avermitilis* and *S. antibioticus* by comparing catechol sensitivity of the wild types and the spontaneous Δ*melC* mutants, *S. avermitilis* B19 and *S. antibioticus* YU5. The result (Fig. 4B) showed that the Δ*melC* mutants were more resistant to catechol. However, in both B19 and YU5, Δ*melC* was part of a large unmapped deletion. To confirm the effect of the Δ*melC* mutation, a complementation test was performed by inserting the *melC* operon into the chromosome of YU5. The *melC^+^* derivative, YU5-117, exhibited similar sensitivity to catechol as the wild type (Fig. 4B, filled triangles). These results confirmed that, in contrast to the protective role of *melD*, *melC* increased the sensitivity of *Streptomyces* to catechol.

Induction of the expression of *melC* requires different amino acids depending on the source of the operon and the hosts [28], [29], [30]. The above tests were performed on R5 medium, which gave strong expression of MelC2 as judged by a high level of melanin production. On minimal medium supplemented with casamino acids and trace elements (MMT), M145/pIJ702-117 produced relatively little melanin, and the culture was only slightly more sensitive to catechol than M145 (Fig. 4C, right panel). On minimal medium without amino acid supplementation (MM), the *melC* operon was not expressed (no melanin production), and there was no difference in catechol sensitivity between M145/pIJ702-117 and M145 (Fig. 4C, left panel). These results supported the role of *melC* in increasing sensitivity to catechol.

The deleterious effects of the deletion of *melD* and the presence of *melC* on the level of phenolic resistance are additive. Introduction of pIJ702-117 into HY69 further increased the sensitivity to catechol (Fig. 5C, open triangles).

### A model is proposed for the opposite effects of *melC* and *melD*

**Figure 6:**
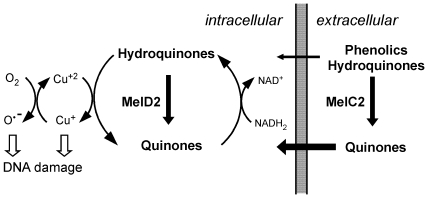
Model for the effect of *melC* and *melD* on sensitivity to phenolic compounds. The intracellular spontaneous quinone-hydroquinone redox cycle is depicted on the left. The proposed oxidation of hydroquinones to quinones by MelD2 is indicated by the vertical thick arrow. Extracellularly, the relative permeability of the hydroquinones/phenolics and the quinones is represented by the thin and thick horizontal arrows, respectively. Conversion of the phenolics/hydroquinones to quinones by MelC2 is indicated by the vertical thick arrow. See text for further details.

To account for the opposite effects of *melC* and *melD* on sensitivity to phenolics, we proposed a working model. The toxicity of the phenolics to *Streptomyces* is at least partly due to their participation in the spontaneous quinone-hydroquinone redox cycle (Fig. 6). In this cycle, copper-catalyzed oxidization of hydroquinones to quinones produces H_2_O_2_ and reactive oxygen species (ROS), which damage DNA [31], [32], [33] in the forms of strand breakage and base modifications [33]. The quinones in turn are reconverted also non-enzymatically by NADH_2_ to hydroquinones. Of the nine phenolics tested here, five were diphenols and triphenols, and presumably may enter the redox cycle readily. The other four (phenol, *o*-aminophenol, ferulic acid, and salicylic acid), being monophenols, presumably were oxidized spontaneously or enzymatically (by MelC2) to diphenols and/or quinones before entering the redox cycle.

We propose that intracellular MelD2 converts hydroquinones to quinones, and, in doing so, reduces or bypasses the spontaneous ROS-generating oxidation catalyzed by Cu^2+^. This would explain the higher phenolic resistance of the *melD* ^+^ strains compared to the *melD* ^−^ strains. The opposite effect by *melC* may be attributed to conversion of the extracellular phenolics (by secreted MelC2) to more hydrophobic quinones, which are taken up by the mycelium more efficiently, and enter the deleterious redox cycle. Although MelC2 is also present intracellularly, it is in an inactive form [5], and presumably has no effect on phenolic toxicity.

### Testing the model (1): The substrate specificity of MelC2 and MelD2 correspond to their selective effects on phenolic sensitivity

The model predicts that MelD2 would oxidize only the phenolics to which the Δ*melD* strain HY69 was supersensitive, and MelC2 would also oxidize the phenolics to which the *melC* ^+^ strains were sensitive. The cytosolic fraction of M145, which did not possess a *melC* operon, was used to test the enzyme activity of MelD2 with the cytosolic fraction of HY69 used as a negative control. For MelC2 enzyme activity, the extracellular fractions of M145/pIJ702-117 and wild-type *S. antibioticus* were tested with those of M145 and YU5 used as the respective negative controls.

Six phenolics were tested spectrophotometrically for oxidization by MelD2 and MelC2. The results (Table 1) showed that MelD2 in M145 cytosol possessed catechol-oxidizing activity (2.6 unit/mg protein). In contrast, the HY69 cytosol contained essentially no activity (<0.04 units/mg protein). Significant oxidation activities by MelD2 were also detected toward the other three compounds, to which HY69 was supersensitive (phenol, 4-methylcatechol, and gallic acid), but not toward caffeic acid and *o*-aminophenol, to which HY69 was not supersensitive. Thus, consistent with our model, all the phenolics that are known to be substrates of MelD2 exerted increased toxicity to the Δ*melD* mutant (Table 2).

**Table 1:** Substrate specificity of MelC2 and MelD2.

	Enzyme activity (units/mg protein)
Strains	Catechol	4-Methylcatechol	Gallic acid	Caffeic acid	*o*-Aminophenol	Phenol
Intracellular (MelD2)^1^						
M145	2.6±0.1	1.2±0.2	1.7±0.3	0.2±0.0	5.9±0.1	0.9±0.1
HY69	0.0±0.0	0.1±0.0	0.4±0.1	0.1±0.0	6.4±0.1	0.4±0.0
Extracellular (MelC2)^2^						
* *M145	0.6±0.1	0.7±0.1	0.8±0.2	0.9±0.1	2.7±0.6	0.5±0.1
* *M145/pIJ702-117	24.4±2.3	13.4±3.3	54.7±5.7	11.1±1.7	60.0±4.5	6.2±0.8
* S. antibioticus* WT	14.0±1.4	11.0±2.6	48.9±6.4	9.3±0.5	64.8±5.4	7.1±0.8
* S. antibioticus* YU5	1.4±0.4	0.7±0.2	1.1±0.2	0.4±0.1	2.3±0.6	1.2±0.2

1 The intracellular proteins in these strains, which did not contain MelC2, were assayed for MelD2 activities.

2 The extracellular proteins in these strains, which did not contain MelD2, were assayed for MelC2 activities.

**Table 2:** Phenolic substrates of MelC2 and MelD2 and effect on toxicity.

	MelC2	MelD2
Compound	Substrate	Increased toxicity	Substrate	Decreased toxicity
Catechol	+^ 1, 2, 4^	+	+	+
Phenol	+ ^2, 4^	+	+	+
4-Methylcatechol	+ ^2, 3^	+	+	+
Gallic acid	+	+	+	+
Caffeic acid	+ ^1^	+	−	−
*o*-aminophenol	+ ^4^	+	−	−
Ferulic acid	N.D.	+	N.D.	±
3,4-Dihydroxybenzoic acid	+ ^1^	+	N.D.	−
Salicylic acid	N.D.	+	N.D.	−

All enzyme activities were determined in this study except for those marked ‘N. D.’ (not determined) and the MelC2 activity on 3,4-dihydroxybenzoic acid, which was based on the reference. References: 1. [34]; 2. [35]; 3. [36]; 4. [37]. ‘±’, slight effect.

The same six phenolics were also tested for substrates for MelC2 in the extracellular fractions of M145/pIJ702-117 and *S. antibioticus*, and the results (Table 1) showed that MelC2 could oxidize all of them, in agreement with previous published results [34], [35], [36], [37]. Another phenolic, 3,4-dihydroxybenzoic acid, which was not tested here, was also reported to be the substrate for MelC2 [34]. All these phenolics showed increased toxicity toward *melC* ^+^ strains (Table 2). These results also supported our model.

### Testing the model (2): Catechol uptake is facilitated by expression of melC

**Figure 7:**
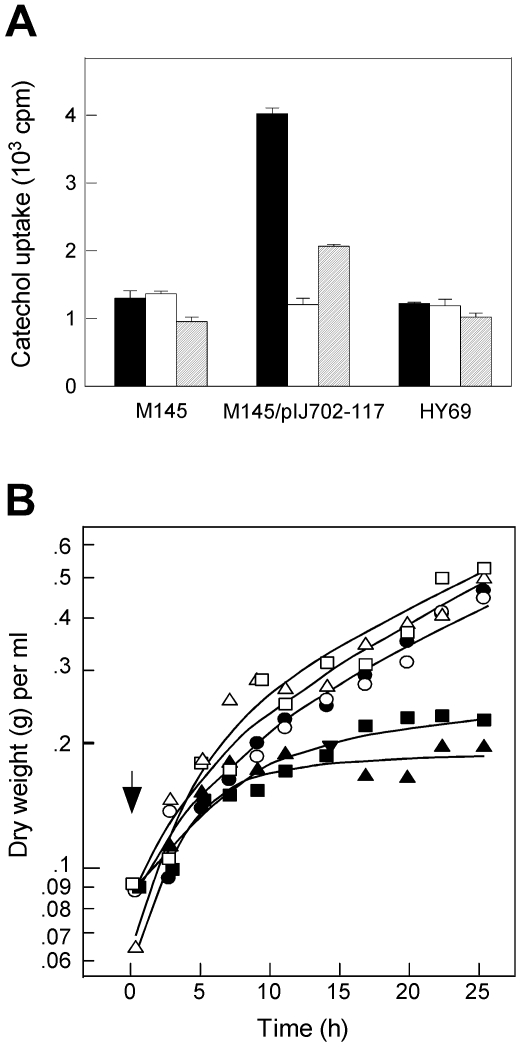
Effect of *melC* and *melD* on catechol uptake by *Streptomyces*. A. Catechol uptake. [^14^C]-catechol was added to a final concentration of 62.5 µM (1.78×10^6^ dpm/µmole) to cultures growing in different liquid media at OD_600_ of 0.4. After 15 min, 20 ml of the cultures was removed and harvested on a filter and radioactivity determined. Media: solid bars, R5; open bars, MM; hatched bars, MMT. The horizontal bars represent the standard deviations. B. Sensitivity of liquid cultures. Catechol was added to a final concentration of 1 mg/ml to the cultures growing in R5 at the time indicated (arrow). At various later times, 3 ml of each culture was removed, and the dry weight was determined by filtration and drying at 60° for 1.5 h. Filled symbols, catechol added; open symbols, no catechol added. Circles, M145; triangles, M145/pIJ702-117; squares, HY69.

Our model also predicts that the presence of functional *melC* facilitates the uptake of catechol into the mycelium. To test this, [^14^C]-labeled catechol was added to M145 and M145/pIJ702-117 growing in R5 liquid medium at late log phase. The M145/pIJ702-117 culture produced melanin in this medium, indicating secretion of active MelC2. After 15 minutes of incubation with catechol, the mycelium was collected by filtration, and the uptake of ^14^C radioactivity was measured by scintillation counting. The results showed that ^14^C radioactivity uptake by M145/pIJ702-117 was about three-fold higher than that by M145 (Fig. 7A, filled bars). Presumably much of the transported ^14^C radioactivity was in the form of 1,2-benzoquinone, which was not available for testing due to its high instability in aqueous solution [38].

When M145 and M145/pIJ702-117 were grown in liquid MM, *melC* was not expressed and there was no significant difference in catechol uptake by the two cultures (open bars). In MMT, *melC* was weakly expressed and catechol uptake by M145/pIJ702-117 was twice that by M145 (hatched bars). These results supported the notion that *melC* expression was essential for increased catechol uptake. HY69 exhibited the same level of catechol uptake in all three media, similar to those exhibited by M145. This indicated that *melD* plays no significant role in catechol uptake.

As on solid R5 medium, growth (measured in dry weight to circumvent the interference of optical density measurement by melanin) of M145/pIJ702-117 and HY69 in liquid R5 medium also showed higher sensitivity to catechol than M145 (Fig. 7B). These results supported the notion that MelC2 increased catechol uptake, resulting in elevated sensitivity.

## Discussion

The PPOs encoded by the *melC*-homologous operons in *Streptomyces* spp. may be classified into three general types (Table 3), each with different cellular locations, enzymatic functions, biological roles, and occurrences. Type I is represented by MelD2, which is mainly membrane bound, and is shown to play a role in protecting *Streptomyces* from damages by a group of phenolics. The *melD* operon is probably universal in *Streptomyces*. Besides our identification of this operon in the ten species, Kirby (2006), using microarray analysis, also identified sequences homologous to *melC1* and *melC2* in three actinomycetes, *Streptomyces cattleya, Streptomyces rimosus*, and *Streptosporangium roseum*. Because these species do not produce melanin, these homologs are likely to be *melD1* and *melD2*. Moreover, Kawamoto *et al.* [24] discovered another *melC-*hybridizing sequence in *melC* deletion (Mel^−^) mutants of *S. lavendulae*. This *melC* homolog is also likely to be a *melD* operon.

**Table 3:** Three types of *Streptomyces* PPOs and tentative distinctions.

Type	Enzyme	Location	Monophenol oxidase activity	Diphenol oxidase activity	*o*-aminophenol oxidase activity	Function	Occurrence
I	MelD2	Intracellular	+	+	−	Detoxification	Universal
II	MelC2	Extracellular	+	+	+	Competition?	Sporadic
III	GriF	Intracellular	−	+	+	Biosynthesis	Rare

Type-II PPOs of *Streptomyces*, represented by MelC2, are found in a proportion of *Streptomyces* species. Unlike the other two types, MelC2 is secreted. Type III has only one example, GriF in *S. griseus* IFO 13550, which is intracellular, and, unlike the other two types, is involved in biosynthesis of a secondary metabolite (grixazone).

In all three types, the gene encoding the PPO is preceded by a gene encoding a helper protein. In *melC*, this helper protein (MelC1) is required for activation (insertion of copper) and secretion of the PPO. It is very likely that the helper protein is also required for activation of the other two types. Interestingly, such action of the helper proteins does not result in secretion of the Type-I and Type-III enzymes. In this study, MelD2 was found to be mostly associated with the membrane. The biochemical and mechanistic significance of this association is not clear.

The three types of PPOs exhibit slightly different substrate specificities (Tables 3, 4). Firstly, while MelC2 and MelD2 possess both monophenol and diphenol oxidase activities that are typical for tyrosinases, GriF lacks the monophenol oxidase activity [15]. Secondly, while GriF [15] and MelC2 [37] oxidize *o*-aminophenols, MelD2 does not oxidize *o*-aminophenol (this study). Thirdly, among these homologs, MelC2 is unique in being able to convert tyrosine to melanin. This may be not surprising, because intracellular oxidization of tyrosine by MelD2 or GriF would probably be detrimental to the cells.

**Table 4:** Bacterial strains and plasmids used in this study.

Designation	Characteristics/genotype	Source/Reference
**A. Bacterial strains**		
* E. coli* ET12567/pUZ8002	Non-methylating plasmid donor for intergeneric conjugation with *Streptomyces*	[60]
* S. antibioticus* IMRU 3720	Wild type	[57]
* *YU5	*ΔmelC* mutant	[10]
YU5-117	YU5 containing pSET152-117 inserted at the ΦC31 *attB* site	This study
* S. avermitilis*	Wild type	Haruo Ikeda
* *B19	*ΔmelC* mutant	This study
* S. coelicolor* A3 (2)		
M145	Prototrophic, SCP1^−^ SCP2^−^	[59]
HY2	M145 with a his6-tagged *melD2*	This study
HY69	*ΔmelD* mutant of M145	This study
* S. lipmanii* ATCC 27357	Wild type	CCRC*
* S. lividans* 1326	Wild type, SLP2^+^, SLP3^+^	[58]
* S. maritimus*	Wild type	[55]
* S. tanashiensis* ATCC 23967	Wild type	[55]
* S. rochei* 7434-AN4	Wild type	[56]
**B. Plasmids**		
pIJ702-117	Derivative of pIJ702 containing *melC* of *S. antibioticus* under a strong promoter	[27]
pSET152-117	Derivative of pSET152 containing *melC* of pIJ702-117	This study
pLUS702melD	pIJ702-117 with *melC* substituted by *melD* and an inserted *aac 3(IV)*	This study
pCR2.1TOPO	*E. coli* cloning vectors, kanamycin and ampicillin resistance, *lacZ*_α_	Invitrogen

* Culture Collection and Research Center (CCRC), Food Industry Research and Development Institute, Hsinchu, Taiwan

*S. griseus* is unusual in containing all three types of *melC*-homologous operons, *melC*, *melD*, and *griEF*. Interestingly, the proteins encoded by all these operons in *S. griseus* exhibit the longest evolutionary distances, and greatest diversification away from the other homologs (Fig. 1C). Moreover, unlike that in the other three sequenced species, *melD* is more distally located than *melC* on the *S. griseus* chromosome, possibly suggesting a relatively recent rearrangement or translocation. The reason for such diverse and rapid evolution of the *melC-*homologous operons in this classical strain [39], which has been studied extensively in laboratories worldwide for more than six decades, is not clear.

Phylogenetic analysis has shown that MelD2s have evolved faster than MelC2s. This is supported by analysis of rates of synonymous and non-synonymous substitution [Ka/Ks analysis; 40]. The average Ka/Ks ratios among the MelC1, MelC2, MelD1, and MelD2 sequences are 0.28±0.09, 0.37±0.02, 0.25±0.03, and 0.24±0.06, respectively. In contrast, the average Ka/Ks ratios between MelC1s and MelD1s and between MelC2s and MelD2s are significantly larger (0.85±0.19 and 0.68±0.09, respectively). Reducing the window size to 50 sites, two segments (aa 30–60 and 130–150) of *S. coelicolor* MelD2 exhibit Ka/Ks ratios reaching 1.4. Superimposing on the tyrosinase structure of *Streptomyces* [41], the first segment contains two of the six histidine residues involved in the two dinuclear copper centers of tyrosinase. The structure of this substrate-binding pocket presumably determines the substrate specificity [41]. Therefore, it is likely that the positive selection exerted here during evolution resulted in alteration of substrate specificity of MelD2.

The opposite effects of MelC2 and MelD2 on the sensitivities of *Streptomyces* to phenolic compounds were initially surprising. In particular, the effect of MelC2 on phenolic sensitivity is opposite to the proposed defense role of this enzyme against plant phenolics [13]. The distinct cellular locations of these two PPOs lead to a biochemical model that account for their opposite effects. This model, that intracellular MelD2 protects the cells by interfering with the spontaneous ROS-generating quinone-hydroquinone redox cycle, and extracellular MelC2 facilitates the uptake of the phenolics by converting them to more hydrophobic quinones, is supported by experimental results reported here.

Why is MelC2 secreted and MelD2 is not? All MelC2 sequences lack a signal peptide. MelC2 of *S. antibioticus* was shown to be secreted *via* the Tat pathway by complexing with MelC1 [6], which contained a signal peptide and a Tat motif [25]. Proteins secreted by the Tat pathway possess a signal peptide and a consensus Tat motif within the signal peptide. Analysis using the Tat signal peptide sequence prediction program TatP [42] revealed a signal peptide and a Tat motif in the MelC1 sequences of eight other species except *S. griseus,* which possess a Tat motif but no signal peptide (Supporting Information, Table S1). This supports the extracellular locations of MelC2 in these species except for *S. griseus*. Of the three *melC* homologous operons in *S. griseus*, *griEF* is not melanogenic. It is not clear whether the *melC* or the *melD* operon is responsible for melanogenesis in *S. griseus*. MelC1 possesses a Tat motif without a signal peptide sequence, and MelD1 possesses a signal peptide sequence without a Tat motif.

Analysis using TatP [42] shows that MelD1 of *S. coelicolor* contained a predicted signal peptide but no Tat motif. This is consistent with the intracellular location of MelD2. The *S. griseus* MelD1 also contains a predicted signal peptide but no Tat motif, and the *S. avermitilis* MelD1 contains neither a signal peptide nor a Tat motif. Thus, these two MelD2 proteins are probably also not secreted. On the other hand, the *S. scabies* MelD1 contains both a predicted signal peptide and a Tat motif. It is possible that this represents an exception for this pathogenic *Streptomyces* or a false-positive prediction by the program. The unavoidable false positives and false negatives in TatP and other related programs [42] demands experimental confirmation for any prediction.

The N-terminal sequences of the MelC1 proteins spanning the signal peptide sequences are relatively conserved, whereas there are wide divergencies among the MelD1 sequences as well as between the MelC1 and MelD1 families. This agrees with the phylogenetic analysis (Fig. 1C), which shows that MelD1 proteins have evolved faster away from MelC1 proteins. The wide divergence of the signal sequences in MelD1 proteins probably reflect evolutionary changes to keep MelD2 intracellular.

Of the five phenolics (catechol, phenol, gallic acid, ferulic acid, and 4-methylcatechol), against which *melD* provides protection, catechol, gallic acid, and ferulic acid are universally present in plants, and *Streptomyces* is expected to be exposed to them frequently in nature. The universal presence of *melD* in *Streptomyces* and its location in the conserved ‘core region’ on most *Streptomyces* chromosomes speak for its biological importance. Against the other four phenolics tested, caffeic acid, 3,4-dihydroxybenzoic acid, *o*-aminophenol, and salicylic acid, *melD* offers no protection. Two other plant phenolics, 3,5-dimethoxy-4-hydroxyacetophenone and catechin dihydrate, did not inhibit the growth of *Streptomyces* strains studied here (disc assay results not shown).

Protective PPO systems are also found in some other bacteria. In *Rhizobium* spp., a ‘tyrosinase’ gene, *melA*, is plasmid-borne. In *R. leguminosarum*, it exists on a ‘symbiotic’ (sym) plasmid together with genes involved in nodulation (*nod*) and nitrogen fixation (*nif*) in the root nodules. In *R. meliloti* GR4, it exists on a non-symbiotic plasmid [43]. These *Rhizobium* tyrosinases, however, are only remotely similar to *Streptomyces* MelC2 in amino acid sequence (about 30% identity) and are considerably larger (609 aas). Unlike the *Streptomyces melC2*/*melD2* genes, *Rhizobium melA* is not associated with a gene (*melC1*/*melD1*) encoding a helper protein.

The *Rhizobium* tyrosinase is intracellular in young cells and produces melanin pigment extracellularly when it is released from the cells (by detergent treatment or autolysis in old colonies). The intracellular location of *Rhizobium* tyrosinase suggests that it plays the same role as *Streptomyces* MelD2 in protecting the cells from damage by phenolics. Consistent with this view, *melA* mutants of *R. etli* are more sensitive to H_2_O_2_, and less efficient in forming nodules [44]. Furthermore, *E. coli* expressing the *R. etli melA* gene exhibits increased resistance to *p*-hydroxybenzoic acid, vanillinic acid, and syringic acids, which are phenolics often present in the soil [44].

The biological function of *melC* in *Streptomyces* is unclear. The substrate for melanin production, tyrosine, is neither the best substrate among the phenolics nor the inducer for *melC* [11]. More likely, *melC* is directed toward toxic phenolics produced by plants rather than tyrosine. In the presence of the toxic phenolics, the detrimental effect it exerts on the hosts can hardly offer a selective advantage. We speculate that the conversion of the phenolics to more permeable quinones by MelC2 is used to fend off other competing soil microbes lacking a protective system like *melD*. This would provide a competitive advantage for *melC* ^+^ *Streptomyces* in the rhizosphere, where phenolics are abundant.

Both phenolics and PPOs are produced by plants and are important defenses against pathogens and pests. Production of PPOs in plants is induced by abiotic and biotic stresses, such as wounds, pathogen infection, and pest invasion [45], [46]. That oxidation of these phenolics by PPO is involved in plant defense was reported recently [47], [48]. Over-expression of PPO in transgenic tomato plants increased resistance to infection by *Pseudomonas syringae* and suppressed growth of the bacterium [48], whereas down regulation of PPO expression by antisense RNA resulted in increased susceptibility to bacterial infection [47]. Several models have been proposed for the changes in susceptibility of infection, and it is possible that a MelC2 scenario is at play here: *i.e.,* the relatively more hydrophobic quinones produced by PPO from the phenolics enter the infecting bacteria more efficiently.

## Materials and Methods

### Bacterial strains and culture conditions

Bacterial cultures and plasmids used in this study are listed in Table 4. DNA restriction, electrophoresis, hybridization, cloning, transformation, and other general biological and molecular procedures were according to Sambrook *et al.* [49] for *E. coli* and Kieser *et al.* [50] for *Streptomyces*. Liquid media MM, MMT, R5 and TSB for *Streptomyces* cultures were described in Kieser *et al*. [50]. MM is minimal medium. MMT is MM supplemented with casamino acids, tyrosine, and ‘Tiger Milk’ (an amino acid cocktail). For dry weight determination, mycelium from 3-ml aliquots was collected on 3MM filter paper, washed with ice-cold water, dried at 60° for 1.5 h, and the dry weights determined.

### Isolation of *melD* operon sequences

Partial *melD* operon sequences were isolated from genomic DNA by PCR using a pair of degenerate primers based on the conserved consensus sequences in the corresponding regions in *melD* of *S. coelicolor*, *S. avermitilis*, *S. scabies,* and *S. griseus* (Forward primer: GACCACTACCGGTCGTACCC[G/C]A; reverse primer: CCAGAACAC[C/G]GGGTCGTTGACG). The PCR products were sequenced.

### Phylogenetic analysis

Amino acid sequences of MelC1/MelD1 and MelC2/MelD2 were aligned using ClustalX version 1.8 [51]. The phylogenetic trees were constructed using the Neighbor-Joining method.

### Creation of *melD* knock-out mutants of *Streptomyces*

Δ*melD* deletion mutant of *S. coelicolor* M145, in which the *melD* operon was replaced by the apramycin resistance gene, *aac 3(IV)*, was created by homologous recombination. *E. coli* plasmid pCR2.1TOPO (Invitrogen) was used to construct a suicide vector that contained a 4.0-kb sequence from *S. coelicolor* spanning the *melD* operon, in which the *melD* operon was replaced by the *aac 3(IV)*-*oriT* cassette [52] (Fig. 3A). This plasmid was transferred into *S. coelicolor* via conjugation with *E. coli* ET12567/pUZ8002 harboring this plasmid. Apramycin resistant and kanamycin sensitive colonies were selected and confirmed by southern hybridization as Δ*melD*::*aac 3(IV)*.

### Assay for catechol uptake by *Streptomyces* cultures

Spores were cultured in R5, MM, and MMT at 30° until the OD_600_ reached 0.4. A 20-ml sample of culture was mixed with 20 µl Cu^2+^ and 1.25 µmole [^14^C]-labeled catechol (80 µCi/nmole) and incubated for 10 min at 30°, and the mycelium was collected on glassfiber filters (Whatman, GF/C), washed with ice-cold water and dried. Radioactivity was measured in a scintillation counter.

### Immunoblot detection of MelC2 and MelD2

MelD2-His6 fusion proteins were detected by immunoblot using monoclonal anti-polyhistidine antibody (Sigma) as the primary antibody and anti-mouse antibody as the secondary antibody. MelC2 proteins were detected by immunoblot using anti-MelC2 antibody as the primary antibody and anti-rabbit antibody as the secondary antibody as described previously [6].

### Determination of sensitivity to phenolic compounds

Dilutions of *Streptomyces* spores were spread on R5 medium containing various concentrations of phenolic compounds. After incubation at 30° for 4 days, the surviving colonies were counted.

### Determination of polyphenol oxidase activity

The PPO activities against phenolics were assayed spectrophotometrically under the following conditions. DOPA: 0.1 M sodium phosphate buffer (pH 7.0), 5 µM CuCl_2_ and 15 mM DOPA at 475 nm. Catechol: 0.1 M sodium phosphate buffer (pH 7.0), 5 µM CuCl_2_ and 4.5 mM catechol at 410 nm. Phenol: 0.1 M sodium phosphate buffer (pH 7.0), 5 µM CuCl_2_ and 2.6 mM phenol at 410 nm [34]. *o*-aminophenol: 50 mM sodium phosphate buffer (pH 7.0), 5 µM CuCl_2_, and 5 mM *o*-aminophenol at 433 nm [15]. 4-methylcatechol: 50 mM sodium phosphate buffer (pH 7.0), 5 µM CuCl_2_, and 4.0 mM 4-methylcatechol at 400 nm [36]. Gallic acid:50 mM sodium phosphate buffer (pH 7.0), 5 µM CuCl_2_, and 7.35 mM gallic acid at 385 nm [modified from 53]. One unit of activity is defined as the amount causing an increase of 0.01 in absorbance per minute.

### Bioinformatic analyses

The presence of Tat signal peptides was predicted using TatP (http://www.cbs.dtu.dk/services/TatP/) [42]. Ka/Ks analysis [40] was performed using DnaSP version 4.0 [54] on a PC running Windows XP.

## Supporting Information

Figure S1 Phylogenetic tree of MelC2 and MelD2 proteins based on the first 310 aas. Filled circles indicate melanin production catalyzed by the enzyme, and open circles indicate lack of melanin production phenotype by the enzyme.
(0.08 MB PDF)

Figure S2 Phylogenetic tree of MelC2 homologs. The following protein sequences are from NCBI annotated databases with accession mumbers. MelC2 from Streptomyces: S. clavuligerus ATCC 27064, ABJH01000362; Streptomyces sp. C (1), ACEW01000543; Streptomyces sp. C (2), ACEW01000650; S. viridochromogenes DSM 40736, ACEZ01000111; S. sviceus ATCC 29083, ABJJ01000408. MelD2 from Streptomyces: S. lividans TK24, ACEY01000212; S. sviceus ATCC 29083, ABJJ01000469; S. viridochromogenes DSM 40736, ACEZ01000014; S. ghanaensis ATCC 14672, ABYA01000366; S. griseoflavus Tu4000, ACFA01000589; Streptomyces sp. SPB78, ACEU01000672; Streptomyces sp. SPB74, ABJG01000510; Streptomyces sp. C (1), ACEW01000540; Streptomyces sp. C (2), ACEW01000097; S. clavuligerus ATCC 27064, ABJH01000487; S. pristinaespiralis ATCC 25486, ABJI01000520; S. rishiriensis MJ773-88K4, ABX71084; S. roseosporus NRRL 11379, ABYX01000271; S. roseosporus NRRL 15998, ABYB01000364; S. tenjimariensis ATCC 31603, CAI59989. Homologs from other bacteria: Rubrobacter xylanophilus DSM 9941, NC_008148; Frankia sp. EAN1pec, NC_009921; Frankia alni ACN14a, NC_008278; Frankia sp. CcI3, NC_007777. The remaining (indicated by an asterisk) are unannotated sequences identified in tblastn search of microbial genomes. The construction of the tree was as described in Fig. 1
(0.14 MB PDF)

Table S1 Predicted Tat signal peptide and motifs in MelC1 and MelD1 proteins based on TatP (version 1.0). * No Tat motif pattern (-RR-X-[FGAVML][LITMVF]) was found, but this similar sequence is present.
(0.07 MB PDF)
