# Supplementary material for: Extracellular and Intracellular Polyphenol Oxidases Cause Opposite Effects on Sensitivity of Streptomyces to Phenolics: A Case of Double-Edged Sword
Source: PLoS One. 2009 Oct 14;4(10):e7462. doi: 10.1371/journal.pone.0007462 (PMC2758597; doi:10.1371/journal.pone.0007462)
Supplement: Figure S1 — Phylogenetic tree of MelC2 and MelD2 proteins based on the first 310 aas. Filled circles indicate melanin production catalyzed by the enzyme, and open circles indicate lack of melanin production phenotype by the enzyme. (0.08 MB PDF) [file pone.0007462.s001.pdf]

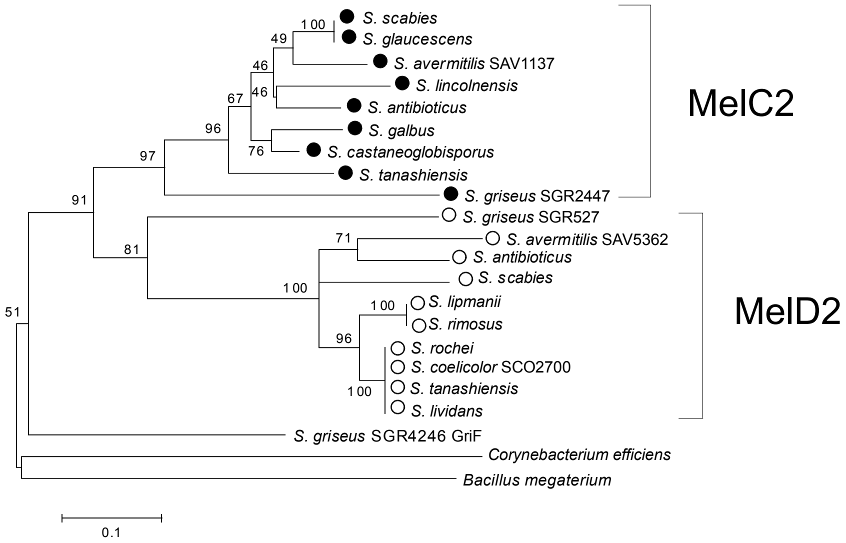

Fig. S1. Phylogenetic tree of MelC2 and MelD2 proteins based on the first 310 aas. Filled circles indicate melanin production catalyzed by the enzyme, and open circles indicate lack of melanin production phenotype by the enzyme.
