# Supplementary material for: Extracellular and Intracellular Polyphenol Oxidases Cause Opposite Effects on Sensitivity of Streptomyces to Phenolics: A Case of Double-Edged Sword
Source: PLoS One. 2009 Oct 14;4(10):e7462. doi: 10.1371/journal.pone.0007462 (PMC2758597; doi:10.1371/journal.pone.0007462)
Supplement: Figure S2 — Phylogenetic tree of MelC2 homologs. The following protein sequences are from NCBI annotated databases with accession mumbers. MelC2 from Streptomyces: S. clavuligerus ATCC 27064, ABJH01000362; Streptomyces sp. C (1), ACEW01000543; Streptomyces sp. C (2), ACEW01000650; S. viridochromogenes DSM 40736, ACEZ01000111; S. sviceus ATCC 29083, ABJJ01000408. MelD2 from Streptomyces: S. lividans TK24, ACEY01000212; S. sviceus ATCC 29083, ABJJ01000469; S. viridochromogenes DSM 40736, ACEZ01000014; S. ghanaensis ATCC 14672, ABYA01000366; S. griseoflavus Tu4000, ACFA01000589; Streptomyces sp. SPB78, ACEU01000672; Streptomyces sp. SPB74, ABJG01000510; Streptomyces sp. C (1), ACEW01000540; Streptomyces sp. C (2), ACEW01000097; S. clavuligerus ATCC 27064, ABJH01000487; S. pristinaespiralis ATCC 25486, ABJI01000520; S. rishiriensis MJ773-88K4, ABX71084; S. roseosporus NRRL 11379, ABYX01000271; S. roseosporus NRRL 15998, ABYB01000364; S. tenjimariensis ATCC 31603, CAI59989. Homologs from other bacteria: Rubrobacter xylanophilus DSM 9941, NC_008148; Frankia sp. EAN1pec, NC_009921; Frankia alni ACN14a, NC_008278; Frankia sp. CcI3, NC_007777. The remaining (indicated by an asterisk) are unannotated sequences identified in tblastn search of microbial genomes. The construction of the tree was as described in Fig. 1 (0.14 MB PDF) [file pone.0007462.s002.pdf]

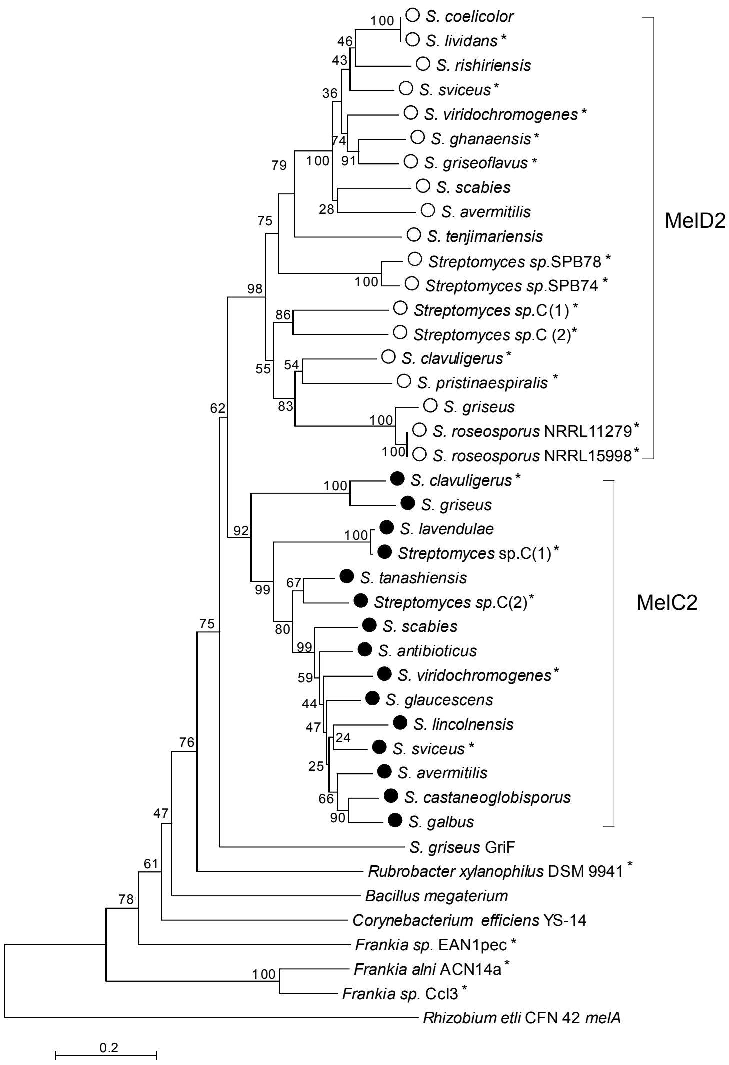

Fig. S2. Phylogenetic tree of MelC2 homologs.

The following protein sequences are from NCBI annotated databases with accession mumbers. MelC2 from *Streptomyces*: *S. clavuligerus* ATCC 27064, ABJH01000362; *Streptomyces* sp. C (1), ACEW01000543; *Streptomyces* sp. C (2), ACEW01000650; *S. viridochromogenes* DSM 40736, ACEZ01000111; *S. sviceus* ATCC 29083, ABJJ01000408. MelD2 from *Streptomyces*: *S. lividans* TK24, ACEY01000212; *S. sviceus* ATCC 29083, ABJJ01000469; *S. viridochromogenes* DSM 40736, ACEZ01000014; *S. ghanaensis* ATCC 14672, ABYA01000366; *S. griseoflavus* Tu4000, ACFA01000589; *Streptomyces* sp. SPB78, ACEU01000672; *Streptomyces* sp. SPB74, ABJG01000510; *Streptomyces* sp. C (1), ACEW01000540; *Streptomyces* sp. C (2), ACEW01000097; *S. clavuligerus* ATCC 27064, ABJH01000487; *S. pristinaespiralis* ATCC 25486, ABJI01000520; *S. rishiriensis* MJ773-88K4, ABX71084; *S. roseosporus* NRRL 11379, ABYX01000271; *S. roseosporus* NRRL 15998, ABYB01000364; *S. tenjimariensis* ATCC 31603, CAI59989. Homologs from other bacteria: *Rubrobacter xylanophilus* DSM 9941, NC_008148; *Frankia sp.* EAN1pec, NC_009921; *Frankia alni* ACN14a, NC_008278; *Frankia sp.* Ccl3, NC_007777. The remaining (indicated by an asterisk) are unannotated sequences identified in tblastn search of microbial genomes. The construction of the tree was as described in Fig. 1.
