# Supplementary material for: Extracellular and Intracellular Polyphenol Oxidases Cause Opposite Effects on Sensitivity of Streptomyces to Phenolics: A Case of Double-Edged Sword
Source: PLoS One. 2009 Oct 14;4(10):e7462. doi: 10.1371/journal.pone.0007462 (PMC2758597; doi:10.1371/journal.pone.0007462)
Supplement: Table S1 — Predicted Tat signal peptide and motifs in MelC1 and MelD1 proteins based on TatP (version 1.0). * No Tat motif pattern (-RR-X-[FGAVML][LITMVF]) was found, but this similar sequence is present. (0.07 MB PDF) [file pone.0007462.s003.pdf]

Table S1. Predicted Tat signal peptide and motifs in MelC1 and MelD1 proteins based on TatP (version 1.0)

Features	Tat signal peptide	Tat motif	Cleavage	Position
MelC1				
S. antibioticus	Y	RRRAL	ARA-DD	30-31
S. avermitilis	Y	RRHAL	ASA-AG	32-33
S. glaucescens	Y	RRRAL	ATA-AG	32-33
S. scabies	Y	RRHAL	AAA-DE	28-29
S. galbus	Y	(RRRAY)*	APA-AT	30-31
S. castaneoglobisporus	Y	RRRAL	ASA-AG	33-34
S. tanashiensis	Y	RRRAL	AVA-AP	31-32
S. lincolnensis	Y	RRRAL	AAA-HD	36-37
S. griseus	N	RRQAL	VPA-GT	35-36
MelD1				
S. avermitilis	N	N	N	N
S. griseus	Y	N	APA-AP	26-27
S. coelicolor	Y	N	AEA-AT	28-29
S. scabies	Y	RRNAL	VVA-AV	40-41
S. griseus GriE	Y	RREMV	EGA-AE	34-35

* No Tat motif pattern (-RR-X-[FGAVML][LITMVF]) was found, but this similar sequence is present.
